# Cellular vaccines in listeriosis: role of the *Listeria* antigen GAPDH

**DOI:** 10.3389/fcimb.2014.00022

**Published:** 2014-02-21

**Authors:** Ricardo Calderón-González, Elisabet Frande-Cabanes, Lucía Bronchalo-Vicente, M. Jesús Lecea-Cuello, Eduardo Pareja, Alexandre Bosch-Martínez, Mónica L. Fanarraga, Sonsoles Yañez-Díaz, Eugenio Carrasco-Marín, Carmen Álvarez-Domínguez

**Affiliations:** ^1^Grupo de Genómica, Proteómica y Vacunas, Primera Planta-Laboratorio 124, Fundación Marqués de Valdecilla-IFIMAVSantander, Spain; ^2^Departamento de Biología Molecular, Facultad de Medicina, Universidad de CantabriaSantander, Spain; ^3^Servicio de Dermatología, Hospital Universitario Marqués de ValdecillaSantander, Spain; ^4^Servicio de Pediatría, Hospital Universitario Marqués de Valdecilla-IFIMAVSantander, Spain; ^5^Information Technologies Research Group, Era7 BioinformaticsGranada, Spain

**Keywords:** dendritic vaccines, *Listeria monocytogenes*, glyceraldehyde-3-phosphate-dehydrogenase

## Abstract

The use of live *Listeria*-based vaccines carries serious difficulties when administrated to immunocompromised individuals. However, cellular carriers have the advantage of inducing multivalent innate immunity as well as cell-mediated immune responses, constituting novel and secure vaccine strategies in listeriosis. Here, we compare the protective efficacy of dendritic cells (DCs) and macrophages and their safety. We examined the immune response of these vaccine vectors using two *Listeria* antigens, listeriolysin O (LLO) and glyceraldehyde-3-phosphate-dehydrogenase (GAPDH), and several epitopes such as the LLO peptides, LLO_189−201_ and LLO_91−99_ and the GAPDH peptide, GAPDH_1−22_. We discarded macrophages as safe vaccine vectors because they show anti-*Listeria* protection but also high cytotoxicity. DCs loaded with GAPDH_1−22_ peptide conferred higher protection and security against listeriosis than the widely explored LLO_91−99_ peptide. Anti-*Listeria* protection was related to the changes in DC maturation caused by these epitopes, with high production of interleukin-12 as well as significant levels of other Th1 cytokines such as monocyte chemotactic protein-1, tumor necrosis factor-α, and interferon-γ, and with the induction of GAPDH_1−22_-specific CD4^+^ and CD8^+^ immune responses. This is believed to be the first study to explore the use of a novel GAPDH antigen as a potential DC-based vaccine candidate for listeriosis, whose efficiency appears to highlight the relevance of vaccine designs containing multiple CD4^+^ and CD8^+^ epitopes.

## Introduction

*Listeria monocytogenes* is a Gram-positive pathogenic bacterium that is widely used as a vector for vaccines against other pathogens or in cancer therapy. However, because it is a human pathogen, it may cause life-threatening infections such as severe meningitis, encephalitis, and brain abscess in pregnant women, neonates, elderly people, and immunocompromised individuals. Vaccination is one of the most successful strategies to treat infectious diseases. However, in the case of listeriosis, there is no vaccine available for high-risk groups such as infants, pregnant women, or individuals with immunological impairment.

Current studies of prophylactic vaccines against *L. monocytogenes* focus on three strategies: (i) the creation of live attenuated pathogens able to access the cytosol and stimulate T cells, including the vaccine vectors with metabolically active but non-viable bacteria; (ii) the use of safe vectors with adjuvant properties able to induce strong immune responses; and (iii) the use of subunit vaccines containing bacterial antigens that might stimulate specific immune responses (Sashinami et al., 2003; Starks et al., 2004; Bruhn et al., 2007; Mohamed et al., 2012; Quispe-Tintaya et al., 2013). There is not a single ideal vector that might be considered safe for human trials, and the use of live vaccines in individuals with any type of immunosuppression poses severe difficulties. Safer vaccines such as subunit vaccines present the disadvantage of requiring strong adjuvants to potentiate their immune responses and these adjuvants might induce undesirable side effects in tissues.

Dendritic cells (DCs) show promise for use as prophylactic or therapeutic vaccine vectors in human trials (Martirosyan et al., 2012; Palucka and Banchereau, 2013; Yu et al., 2013). DCs play a major role in the development of cell-mediated immunity because they link innate and adaptive immune responses. They are powerful antigen-presenting cells (APCs) and have a distinct ability to prime naïve T helper (Th) T lymphocytes and cytotoxic T lymphocytes (CTLs). These features of DCs are responsible for their immunostimulatory potential. In fact, they have been used as cellular vaccines or adjuvants in immunotherapy against cancer or infections, such as listeriosis or aids (Pion et al., 2010; Kono et al., 2012; Vacas-Cordoba et al., 2013).

Macrophages are also efficient APCs and crucial cells for the innate and adaptive immune responses against listeriosis (Ziegler and Unanue, 1981; Portnoy et al., 2002; Pamer, 2004). In fact, *L. monocytogenes* natural habitats are the monocytes and macrophages, therefore, it seems likely that using these cells loaded with the pathogen might secrete unique antigens and generate protective immune responses. Macrophages infected with other bacteria such as *Salmonella typhimurium* or *Mycobacterium tuberculosis* have been successfully used as safe vaccines, conferring good protection against these pathogens (Sharma and Agrewala, 2004; Singh et al., 2011).

Murine *L. monocytogenes* infection is characterized by the development of a protective T-cell-dependent immunity mediated by both CD4 and CD8 T cells. Several T cell immunodominant epitopes restricted by MHC-I molecules have been reported such as the listeriolysin O (LLO) epitope LLO_91−99_ and the p60 epitope p60_217−225_ (Sijts et al., 1996; Pamer, 2004). Less information is available concerning immunodominant CD4 epitopes restricted by MHC-II molecules, because only LLO epitope LLO_189−201_ appears as immunodominant and p60 epitope p60_301−312_ as subdominant (Geginat et al., 2001). However, several *other L. monocytogenes* virulence factors such as ActA, MLP or glyceraldehyde-3-phosphate-dehydrogenase (GAPDH) (Álvarez-Domínguez et al., 2008) appear to induce strong immune responses, and therefore, they might contain putative epitopes that confer protection against listeriosis. In this study, we examined the ability of a novel GAPDH peptide, GAPDH_1−22_ to confer protection against listeriosis when incorporated into macrophages or DCs as vaccine vectors. Our data showed that macrophages infected with *L. monocytogenes* were unsafe vaccine vectors, while GAPDH_1−22_-loaded DCs were safe and more efficient vaccine vectors against listeriosis than LLO_91−99_-loaded DCs.

## Methods

### Animals

We used C57BL/6 mice from our animal facilities at the University of Cantabria. Bone-marrow-derived macrophages (BMDMs) or bone-marrow-derived DCs were obtained from femurs of 8–12-week-old female mice. BMDMs or DCs were cultured at 2 × 10^6^ cells/ml in six-well plates in Dulbecco's Modified Eagle's Medium (DMEM) supplemented with 20% fetal calf serum (FCS), 1 mM glutamine, 1 mM non-essential amino acids, 50 μg/ml gentamicin and 30 μg/ml vancomycin (DMEM complete medium) and 25 ng/ml macrophage colony-stimulating factor (M-CSF) for BMDMs or 25 ng/ml granulocyte–macrophage colony-stimulating factor (GM-CSF). On Day 7, the cells were harvested and analyzed by fluorescence-activated cell sorting (FACS) to evaluate cell surface markers and appropriate differentiation of BMDMs or DCs using the following markers: CD11b–fluorescein isothiocyanate (FITC), CD11c–phycoerythrin (PE), IA^b^–APC, F4/80–PE, CD80–FITC, and CD86–V450. Cells were collected using cell scrapers for detaching adherent cells. In certain samples we also used after detaching adherent cells positive selection using anti-mouse CD11c-coated magnetic beads and MACSTM separation columns (Miltenyi Biotech Inc., Auburn, CA) on Day 7 as previously described (Kono et al., 2012).

### Bacteria

*L. monocytogenes* 10403S strain (LM-WT) and the *hly*-deficient LM mutant (LM-ΔLLO, DPL-2161 strain) were obtained from D. A. Portnoy (University of California, Berkeley, CA, USA) and green fluorescent protein (GFP)–*L. monocytogenes* variant of the LM strain DH-L1039 (GFP-LM) was kindly provided by D. E. Higgins (Harvard Medical School, Boston, MA, USA).

### Kinetic infection assays

Differentiated BMDMs or DCs were cultured in 96-well plates at 1 × 10^6^ cells/ml and infected with LM-WT or LM-ΔLLO at a ratio of 10: 1 (bacteria: cells) as previously reported for different times (0, 4, 8, or 16 h) (Prada-Delgado et al., 2001). CFU ratios are performed as reported and represented the ratio of CFU at 8 h to CFU at 0 h ± *SD* of triplicates (Prada-Delgado et al., 2001; Carrasco-Marín et al., 2009).

### Postnuclear supernatant isolation and immunoprecipitation

BMDMs and DCs were non-infected (NI) or infected with *L. monocytogenes* for 20 min (10:1; bacterium: cell ratio) and cells were homogenized in HBE buffer and postnuclear supernatants (PNSs) were obtained as previously reported (Álvarez-Domínguez and Stahl, 1999, Prada-Delgado et al., 2001; Del Cerro-Vadillo et al., 2006; Carrasco-Marín et al., 2009, 2011, 2012). Total membranes were pelleted from thawed PNSs after 35,000 × *g* ultracentrifugation, as previously described (Carrasco-Marín et al., 2012). To detect LLO bound to MHC class II molecules, total membranes from PNSs were immunoprecipitated with mouse anti-IA^b^ antibody (Y3P). Immunoprecipitates were run onto SDS-PAGE, transferred to nitrocellulose membranes and incubated with primary antibodies, rabbit anti-LLO or rabbit anti-GAPDH_1−22_ and horseradish-peroxidase-conjugated secondary antibodies.

### Confocal microscopy

BMDMs or DCs used for immunocytochemistry were cultured in 24-well plates with coverslips at 1 × 10^6^ cells/ml and infected with GFP-LM at a ratio of 10: 1 (bacteria: cells) for 1 h. Cells were washed with PBS and fixed in 3% paraformaldehyde. BMDMs or DCs were labeled with biotinylated anti-IA^b^ antibody (40F monoclonal antibody) followed by TRICT-streptoavidin, CD11c–PE or CD11b–PE, as previously described (Rodriguez-Del Rio et al., 2011; Frande-Cabanes et al., 2014). All confocal procedures with antibodies included a permeabilization treatment with PBS-0.05% Triton X-100 as previously reported (Carrasco-Marín et al., 2012). Confocal microscopy imaging was performed with a Nikon A1R confocal microscope equipped with the laser lines: 405, 488, 514, 561, 638 nm using 60× Plan Apochromat VC 1.4 NA oil objective.

### Peptides

We used the following peptides, GAPDH_1−22_, LLO_1−99_, and LLO_189−201_, the latter includes all MHC-II epitopes of LLO reported for IA^b^ mouse haplotype, LLO_189−200_ and LLO_190−201_ (Ziegler and Unanue, 1981; Sijts et al., 1996; Geginat et al., 2001; Skoberne and Geginat, 2002; Carrero et al., 2012). All peptides were synthesized and purified by *F. Roncal* (CNB. CSIC. Madrid) after HPLC and Mass Spectrometry using a MALDI-TOF Reflex™ IV Bruker (Bruker Daltonics, Bremen, Germany) mass spectrometer. Peptide synthesis showed purity >95% after HPLC and mass spectrometry.

### Preparation of peptide-pulsed BMDMs or DCs

Differentiated and mature BMDMs or DCs were cultured in DMEM complete medium at a concentration of 2 × 10^6^ cells/ml and pulsed with 5 μM LLO_91−99_, LLO_189−201_, or GAPDH_1−22_ for 24 h. Cells were washed twice in Hank's Balanced Salt Solution (HBSS), detached with cell scrapers and counted.

### Vaccination protocols

Differentiated and matured BMDMs or DCs were cultured in six-well plates at 2 × 10^6^ cells/ml and incubated with different peptides, as described in the previous section. For BMDMs or DCs loaded with LM-WT vaccines, we infected cells with *L. monocytogenes* as above for 0 h. Cells were washed and cultured in DMEM–2% FCS and 20 μg/ml gentamicin for 24 h. Cells were washed with HBSS, detached with cell scrapers, washed three times, counted, and cell pellets were resuspended at 1 × 10^6^ cells/ml in DMEM–20 μg/ml gentamicin. Different BMDM or DC vaccine vectors (5 × 10^5^ cells/mice) were inoculated in the peritoneal cavity of mice (*n* = 5) for 7 days or were non-vaccinated (NV). In same controls, vaccinations were performed with peptides in solution (5 μM) administrated similar to DC vaccines. All mice were inoculated with 10^3^ CFU *L. monocytogenes* i.p. for an additional 3 days. The vaccination timing followed similar protocols reported for studies using *L. monocytogenes* as a vaccine (Lauer et al., 2008; Rodriguez-Del Rio et al., 2011; Carrasco-Marín et al., 2012; Kono et al., 2012). Mice were bled before sacrifice and serum stored at −80°C to measure cytokines by FACS analysis. Spleens and livers were homogenized and CFUs counted in homogenates.

### FACS analysis of BMDMs, DCs, and cytokine measurements

BMDMs or DCs were cell surface labeled with antibodies against the following markers: CD11b (for macrophages), CD11c (for DCs), F4/80 (for activated phagocytes), CD80 (for mature DCs), CD86 (for mature DCs) or IA^b^ (MHC-II for C57BL/6 mice) and analyzed by FACS. Cytokines of vaccinated and NV mice were quantified by using the CBA kit (Becton Dickinson, Palo Alto, CA, USA). Samples were performed in triplicate and the results are the mean ± *SD* of two separate experiments. ANOVA was used for statistics with the cytokine measurements. Data were analyzed using the FlowJo software (Treestar, Ashland, OR).

### FACS analysis of spleens

We used FACS analysis for cell surface labeling of spleen cells (10^5^ cells), using monoclonal antibodies FITC or APC-labeled (BD Biosciences, San Jose, CA, USA) against CD4 or CD8. Peptides used were LLO_1−99_, LLO_189−201_, or GAPDH_1−22_. For measuring intracellular IFN-γ, spleen cells were plated into 96-well round-bottom plates (5 × 10^6^ cells/ml) and stimulated with each of the LLO_91−99_, LLO_189−201_, or GAPDH_1−22_ peptides independently (10^−5^ M each peptide) for 5 h in the presence of brefeldin A (intracellular cytokine staining), as described previously (Bahjat et al., 2008; Lauer et al., 2008; Rodriguez-Del Rio et al., 2011; Carrasco-Marín et al., 2012). Stimulated cells were surface stained for CD4 and CD8 and then fixed and permeabilized using a cytofix/cytoperm kit to measure intracellular IFN-γ (BD Biosciences). Samples were acquired using a FACSCanto flow cytometer (BD Biosciencies). Data were gated to include exclusively CD4^+^ or CD8^+^ events, and the percentages of these cells expressing IFN-γ were determined according with the manufacturer's recommendations. Results of LLO-peptide-stimulated splenocytes were corrected according to the percentages of total CD4^+^ and CD8^+^ cells. Data were analyzed using FlowJo software (Treestar, Ashland, OR, USA). To confirm the frequency of LLO_91−99_- or GAPDH_1−22_-specific CD8 T cells producing IFN-γ, we used recombinant soluble dimeric mouse H-2K^b^:Ig fusion protein following the instructions of the manufacturer (DimerX I; BD Bioscience). LLO_91−99_ or GAPDH_1−22_ peptides (40 μM) were preincubated with H-2K^b^:Ig (1 μM) in PBS, at 37°C for 16 h and incubated with the staining cocktail mix containing PE-conjugated A85-1 mouse monoclonal antibody (BD Biosciences) for 1 h at room temperature. Splenocytes (2 × 10^7^ cells/ml) were incubated with IFN-γ and CD8 antibodies and the staining cocktail mix described above for 10 min at 4°C. Percentages of CD8^+^ gated cells were expressed as the mean ± *SD* of triplicates (*P* < 0.05). Data were analyzed using FlowJo software.

### Statistical analysis

For statistical analysis, the Student's *t*-test was applied. ANOVA analysis was applied also for cytokine measurement. *P* ≤ 0.05 was considered significant. GraphPad software was used for graphic presentation.

### Ethics statement

This study was carried out in strict accordance with the recommendations in the Guide for the Care and Use of Laboratory Animals of the Spanish Ministry of Science, Research and Innovation. The Committee on the Ethics of Animal Experiments of the University of Cantabria approved this protocol (Permit Number: 2012/06) that follows the Spanish legislation (RD 1201/2005). All surgery was performed under sodium pentobarbital anaesthesia, and all efforts were made to minimize suffering.

## Results

### Preparation of BMDM and DC vaccine vectors infected with LM

To use BMDMs and DCs as vectors loaded with pathogenic *L. monocytogenes*, we first discarded any differences in BMDM and DC phagocytic parameters, balancing their differentiation and maturation states. We differentiated both cells with their corresponding growth factors, M-CSF or GM-CSF and checked their maturation states. All BMDM preparations were CD11b^+^F4/80^low^IA^b+^CD45^+^ with 90% double-positive CD11b^+^CD45^+^ and 60% positive IA^b+^ cells by FACS. DC preparations were CD11c^+^F4/80^low^CD11b^low^IAb^+^ with 95% positive CD11c^+^ and 70% positive IA^b+^ cells.

**Figure 1:**
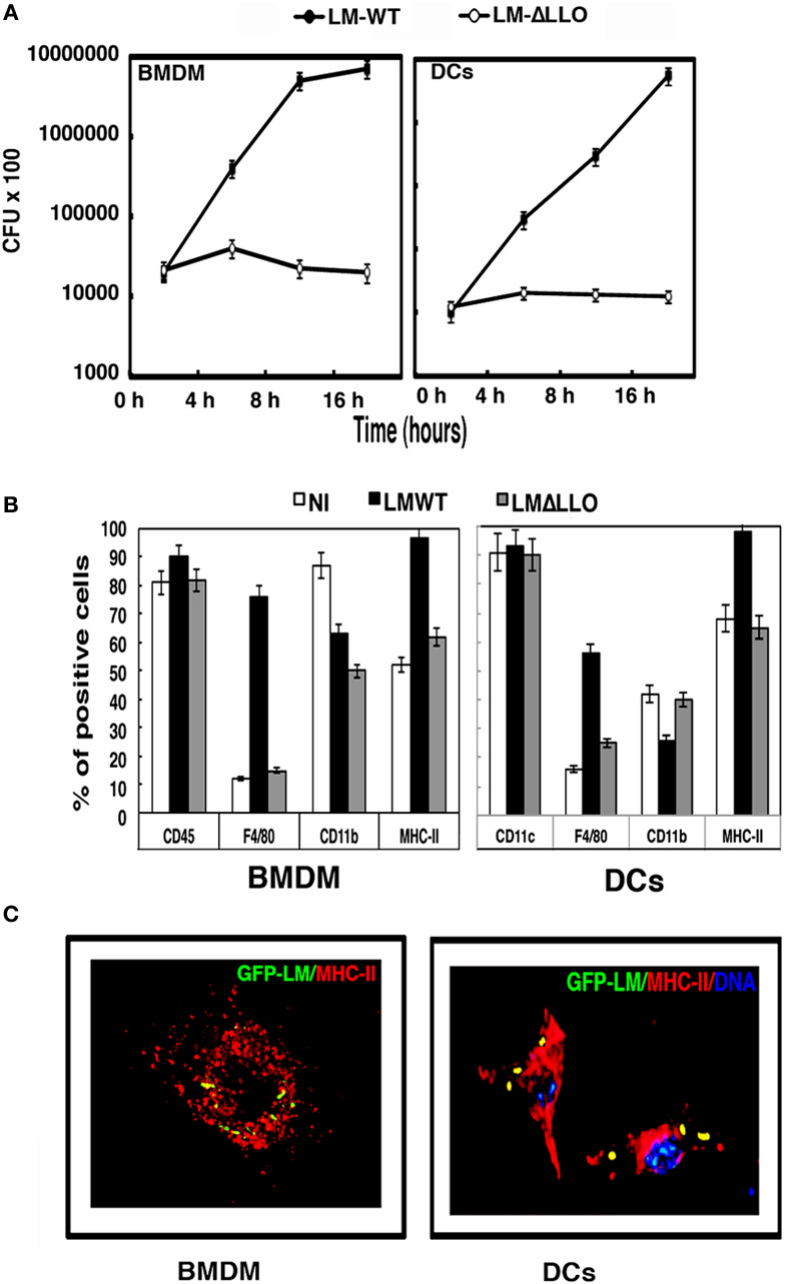
**BMDM and DC vaccines infected with different *L. monocytogenes* strains. (A)** Kinetic analysis of murine BMDMs and DCs infected with LM-WT or LM-ΔLLO. Results are expressed as CFU (mean ± *SD*) obtained with triplicate samples from three independent experiments (main differences are observed between LM-WT and LM-ΔLLO results, *P* < 0.05). **(B)** BMDMs or DCs were infected with LM-WT or LM-ΔLLO (10:1 ratio of bacteria: cells) for 2 h or were non-infected (NI), detached from plates, washed several times, and surface stained with the following FITC- or PE-labeled antibodies: CD45–FITC, F4/80–PE, CD11b–APC, CD11c–PE, or anti-IA^b^–APC. Samples were acquired using a FACSCanto flow cytometer and percentages of positive cells for each antibody are shown. Results are expressed as the mean ± *SD* of triplicate samples (*P* < 0.05). **(C)** Images correspond to confocal microscopy projections of BMDMs or DCs infected with GFP-LM (green channel), anti-MHC-II antibodies that label the antigen-processing compartments (Y3P biotinylated antibody followed by goat anti-mouse TRITC labeled) (red channel) and DNA (blue channel). Colocalization of GFP-LM and MHC-II (yellow fluorescence) is observed in BMDMs and DCs images. All BMDM preparations were CD11b^+^CD45^+^F4/80^high^MHC-II^high^, reflecting their macrophage origin and purity and DC preparations were CD11c^+^F4/80^high^CD11b^low^MHC-II^high^, reflecting their DC origin and purity.

We examined whether *L. monocytogenes* was able to infect both cells with similar kinetics. Infection of BMDMs with pathogenic *L. monocytogenes* (LM-WT) showed a characteristic exponential proliferation of 4 h duration that ended in a plateau phase of growth at 16 h post-infection. However, LM-WT growth in DCs remained exponential even at 16 h post-infection (Figure 1A, black circles). Listericidal phagocytes degraded *L. monocytogenes* within the phagosomal environment.

To compare the listericidal abilities of both BMDMs and DCs, we used a *L. monocytogenes hly*-deficient strain (LM-ΔLLO) with a known gene deletion that rendered it unable to escape from phagosomes. BMDMs and DCs had similar listericidal activity, because LM-ΔLLO strain did not replicate in BMDMs or DCs (Figure 1A, white circles). Activation of phagocytes seems to be a prerequisite to trigger a powerful and protective immune response against *L. monocytogenes*. High percentages of positive MHC-II^+^ and F4/80^+^ cells are characteristics of activated phagocytes. Infection with LM-WT increased the number of positive cells for MHC-II and F4/80 in BMDMs and DCs, showing 95–100% positive IA^b^ cells in both cell types (Figure 1B, white bars). However, the number of positive cells of markers not involved in activation such as CD11b for BMDMs or CD11c for DCs was not increased but diminished (Figure 1B, black bars). LM-ΔLLO strain infection caused no modification in the number of positive MHC-II^+^ or F4/80^+^ BMDMs or DCs (Figure 1B, gray bars). In summary, the phagocytic features of BMDMs and DCs infected with LM-WT appeared similar but clearly different than when these cells were infected with LM-ΔLLO. This prompted us to discard the use of LM-ΔLLO mutants in cellular vaccination.

We measured the ability of phagosomes to transform into antigen-processing compartments or MIIC using a method that colocalized GFP-LM-WT with MHC-II molecules in BMDMs and DCs infected with *L. monocytogenes* (Carrasco-Marín et al., 2011). Analysis of confocal images indicated significant colocalization of GFP-LM with MHC-II molecules in BMDMs and DCs (yellow fluorescence in Figure 1C). CD11b or CD11c molecules, not involved in antigen presentation and in the case of CD11c with a preferential surface pattern, did not colocalize with GFP-LM (data not shown). BMDMs and DCs showed similar GFP-LM colocalization with MHC-II molecules, 70 and 72% rates of colocalization, respectively. In summary, BMDMs infected with LM-WT showed similar phagocytic, activation and antigen processing features as DCs.

### Selection of *L. monocytogenes* antigens to evaluate primary T cell responses after vaccination with BMDMs and DCs

**Figure 2:**
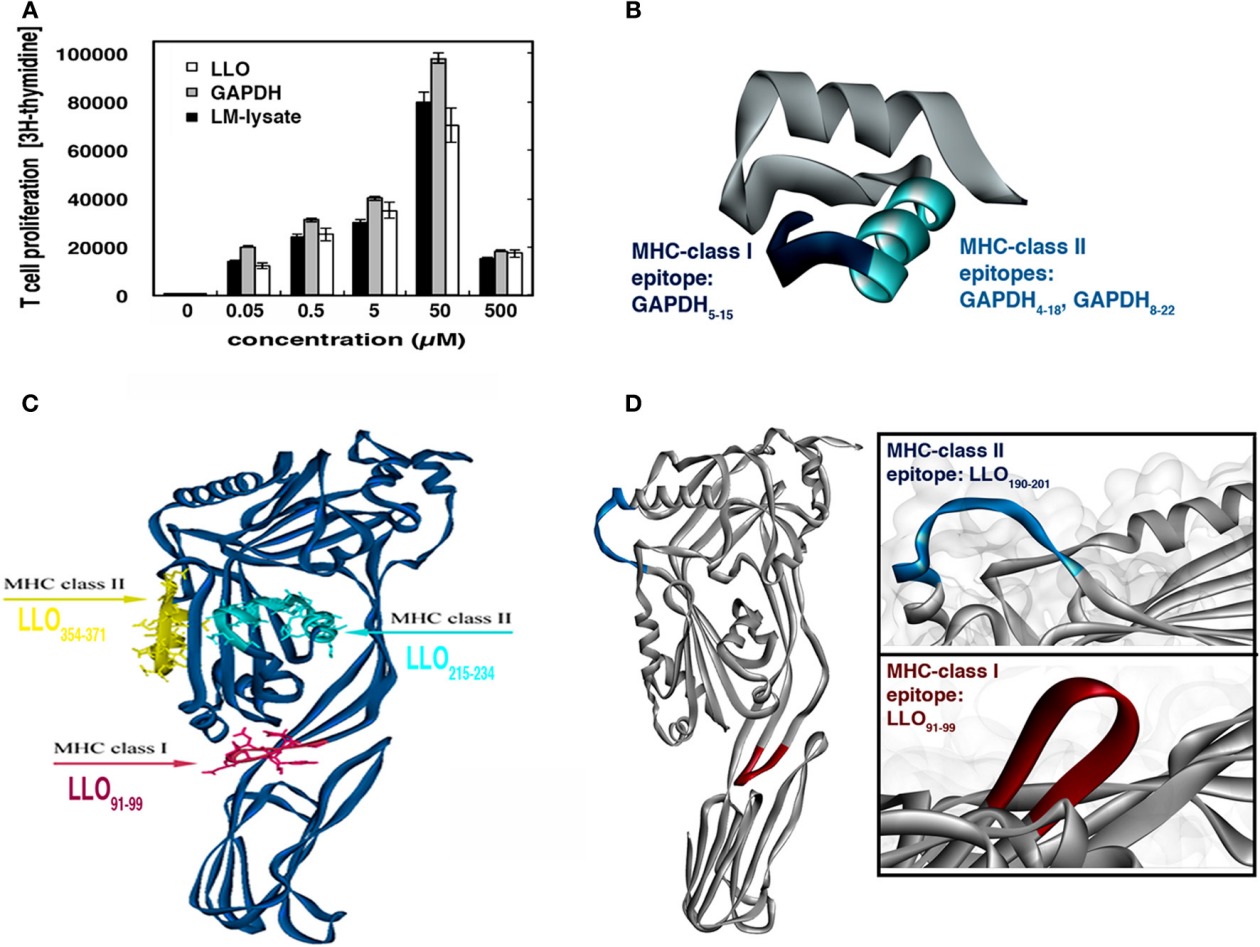
**Selection of *L. monocytogenes* antigens to evaluate primary T cell responses after vaccination with BMDMs and DCs. (A)** Thirty micrograms of total membranes of BMDMs or DCs infected *with L. monocytogenes* was immunoprecipitated with mouse anti-MHC-II, and subjected to western blotting with rabbit anti-LLO (upper lanes) or rabbit anti-GAPDH_1−22_ (lower lanes). NI lanes correspond to non-infected BMDMs or DCs. **(B)** T cell proliferation of homogenates of popliteal lymph nodes after hind foot pad inoculation with 30 μg LM-lysate. Cells were stimulated with LLO, GAPDH, or LM-lysate. Results show [^3^H]-thymidine incorporation in triplicate samples of T cells. **(C)** 3D model of GAPD_1−22_ peptide showing the MHC-I binding sequences in pink and the MHC-II binding sequences in yellow. **(D)** 3D structure of LLO showing the MHC-I and MHC-II epitopes in the C57BL/6 mouse model.

LLO appears as the immunodominant antigen that elicits specific T cell responses in listeriosis (Geginat et al., 2001; Skoberne and Geginat, 2002; Pamer, 2004; Bruder et al., 2005; Carrero et al., 2012). Studies from our group have also characterized a novel *L. monocytogenes* antigen, GAPDH that generates peptide-restricted antibodies in the absence of adjuvants, suggesting the induction of strong T cell responses (Álvarez-Domínguez et al., 2008). First, we verified that LLO and GAPDH generated specific T cells responses with similar efficiencies. Macrophages and DCs are the main APCs in lymph nodes able to stimulate T cells. We immunized the hind footpads of mice with a bacterial lysate (LM-lysate) in the absence of adjuvants as described previously (Mohamed et al., 2012) and recovered the popliteal lymph nodes to evaluate specific T cell responses to different antigens *in vitro*. T cell responses were dose dependent, similar for purified LLO, GAPDH *or L. monocytogenes* lysates, and had a significant peak at 50 μM concentrations (Figure 2B). Therefore, immunization with bacterial lysates generated LLO- and GAPDH-specific T cells in similar ranges. These results strongly suggested that macrophages and DCs *in vivo* processed LLO and GAPDH with similar efficiencies, since their T cell responses were comparable or even slightly enhanced with GAPDH (gray bars in Figure 2A). We also confirmed antigen processing efficiency after immunoprecipitation of MHC-II molecules from total membranes of BMDM and DCs infected with LM-WT. We recovered LLO and GAPDH bound forms to MHC-II molecules as previously reported (Carrasco-Marín et al., 2012) (data not shown).

We compared the surface expression patterns of BMDMs or DCs loaded with purified LLO or GAPDH, or after infection with LM-WT. We prepared BMDMs and DCs followed by infection with LM-WT (bacteria: cells ratio, 10:1) (BMDM-LM-WT or DC-LM-WT) or loaded with 50 μM purified LLO (BMDM-LLO or DC-LLO) or GAPDH (BMDM-GAPDH or DC-GAPDH) for 16 h. BMDM-LLO, DC-LLO, BMDM-GAPDH, and DC-GAPDH showed an increase in the percentages of MHC-II^+^ positive cells, similar to the enhancement observed with DC-LM-WT. However, DC-LLO or DC-GAPDH caused no significant modification in the numbers of positive CD11b^+^ or CD11c^+^ cells (Table 1). These data suggest that DC-LM-WT, DC-LLO, and DC-GAPDH showed similar percentages of positive MHC-II^+^ cells that might correspond to mature DCs and not to immature DC. Incubation of DC with certain peptides was previously reported to cause similar DC maturation (Pion et al., 2010; Kono et al., 2012).

**Table 1:** **Surface markers of different BMDM and DC vaccine vectors**.

	**BMDM^1^**		**DC**	
	**CD11b**	**MHC-II**	**CD11c**	**MHC-II**
NI	87 ± 1.5	62 ± 1.2	87 ± 1.3	72 ± 1.3
LM-WT	73 ± 1.3	90 ± 2.0	98 ± 1.5	92 ± 1.4
LLO	70 ± 1.2	92 ± 1.9	87 ± 1.6	92 ± 1.5
GAPDH	73 ± 1.3	92 ± 1.8	89 ± 1.7	92 ± 1.6

BMDMs and DCs were infected with LM-WT (10:1 ratio of bacteria: cells) or incubated with 50 μg/ml purified LLO or GAPDH for 16 h, detached from the plates, washed several times, and surface stained with the following FITC- or PE-labeled antibodies: CD11b–FITC, CD11c–PE, anti-IA^b^–APC. Samples were acquired using FACSCanto flow cytometer and percentages of positive cells for each antibody are shown. Results are expressed as the mean ± *SD* of triplicate samples.

1 *(P < 0.05)*.

Widely characterized LLO immunodominant CD8^+^ and CD4^+^ epitopes are: CD8^+^-restricted LLO_91−99_ peptide that binds to MHC-I molecules (Kb, Kd, or Ld) and CD4^+^-restricted LLO_189−201_ peptide that binds to MHC-II molecules (IA^b^) (Geginat et al., 2001; Skoberne and Geginat, 2002; Bruder et al., 2005). No information on GAPDH immunodominant CD4^+^ or CD8^+^ epitopes is available, therefore, we performed prediction analysis on the binding abilities of GAPDH_1−22_ to MHC-I or MHC-II molecules using a prediction method with the IEDB analysis resource Consensus tool (Kim et al., 2012), which combines predictions from ANN (Nielsen et al., 2003; Lundegaard et al., 2008), SMM (Peters and Sette, 2005), and Comblib (Sidney et al., 2008). We also included in this analysis LLO_91−99_ and LLO_189−201_ peptides because these epitopes were eluted from MHC-I and MHC-II molecules, respectively, and their binding affinities are known (Geginat et al., 2001; Skoberne and Geginat, 2002) (Table 2). IEDB analysis predicted that good binders showed percentile ranks <10, while weak binders showed percentile ranks <100. GAPDH_1−22_ peptide was a strong binder to MHC-I molecules, similar to LLO_91−99_. The following GAPDH sequences showed the highest affinities, GAPDH_5−15_ with a 1.60 percentile rank and GAPDH_8−18_ with a 2.3 percentile rank, compared with a 1.80 percentile rank for LLO_91−99_ (Table 2, MHC-I binding values). With regards to binding to MHC-II molecules, it seems that GAPDH_1−22_ peptide and LLO_189−201_ were weak binders, with percentile ranks of 74. The GAPDH sequence with the lower percentile ranks was GAPDH_4−18_ with 69.15. GAPDH_8−22_ showed percentile ranks similar to LLO_189−201_ (Table 2, MHC-II binding values). When we applied these data to the bioinformatics modeling of GAPDH_1−22_ and LLO 3D structures (Figures 2C,D), LLO_189−201_ presented an α-helix type 3D structure, while LLO_91−99_ peptide had a loop structure (Figure 2D). GAPDH_5−15_ also appeared as a loop 3D structure (Figure 2C, pink in 3D model) and GAPDH_8−22_ showed an α-helix 3D structure (Figure 2C, yellow in 3D model). The theoretical 3D predictive model for *Listeria monocytogenes* GAPDH was produced using the Automated Comparative Protein Modeling Server SWISS-MODEL available at: (http://www.expasy.ch/swissmod/SWISS-MODEL.html). Therefore, it seems that GAPDH_1−22_ peptide might contain at least two MHC-I binding sequences as well as two MHC-II binding sequences in the C57BL/6 mouse model. Taken together, these results indicate that LLO and GAPDH, as well as their epitopes, are good candidates to include in cellular vaccine designs against listeriosis.

**Table 2:** **MHC-I and MHC-II binding force predictions for LLO and GAPDH epitopes**.

**MHC-I binding**	**MHC-I allele**	**Percentile rank**	**Sequence**
LLO_91−99_	H-2-Kd	1.8^*^	Complete (GB)
	H-2-Ld	38.3	
	H-2-Kb	75.1	
GAPDH_1−22_	H-2-Kb	1.6^*^	5–15^*^
	H-2-Ld	2.3	8–18
	H-2-Kd	4.7	8–16
**MHC-II binding**	**MHC-II allele**	**Percentile rank**	**Sequence**
LLO_190−201_	H-2-IAb	74.3^*^	Complete (WB)
GAPDH_1−22_	H-2-IAb	69.15^*^	4–18^*^
	H-2-IAb	70.23	5–19
	H-2-IAb	70.91	2–16
	H-2-IAb	71.11	3–17
	H-2-IAb	74.95^*^	8–22^*^
	H-2-IAb	75.61	7–21
	H-2-IAb	75.72	1–15

*Predictions of binding of peptides to MHC molecules performed with the IEDB analysis source Consensus tool. The lower the percentile ranks obtained, the better the binders. GAPDH_1-22_ peptide was compared to the predictions of LLO_91-99_ binding to MHC-I molecules and LLO_189-201_ binding to MHC-II molecules*.

* *Comparative peptide sequence with similar binding percentiles as LLO peptides. GB, good binder; WB, weak binder*.

### Comparison of different BMDM and DC vaccine vectors for protection against listeriosis

**Figure 3:**
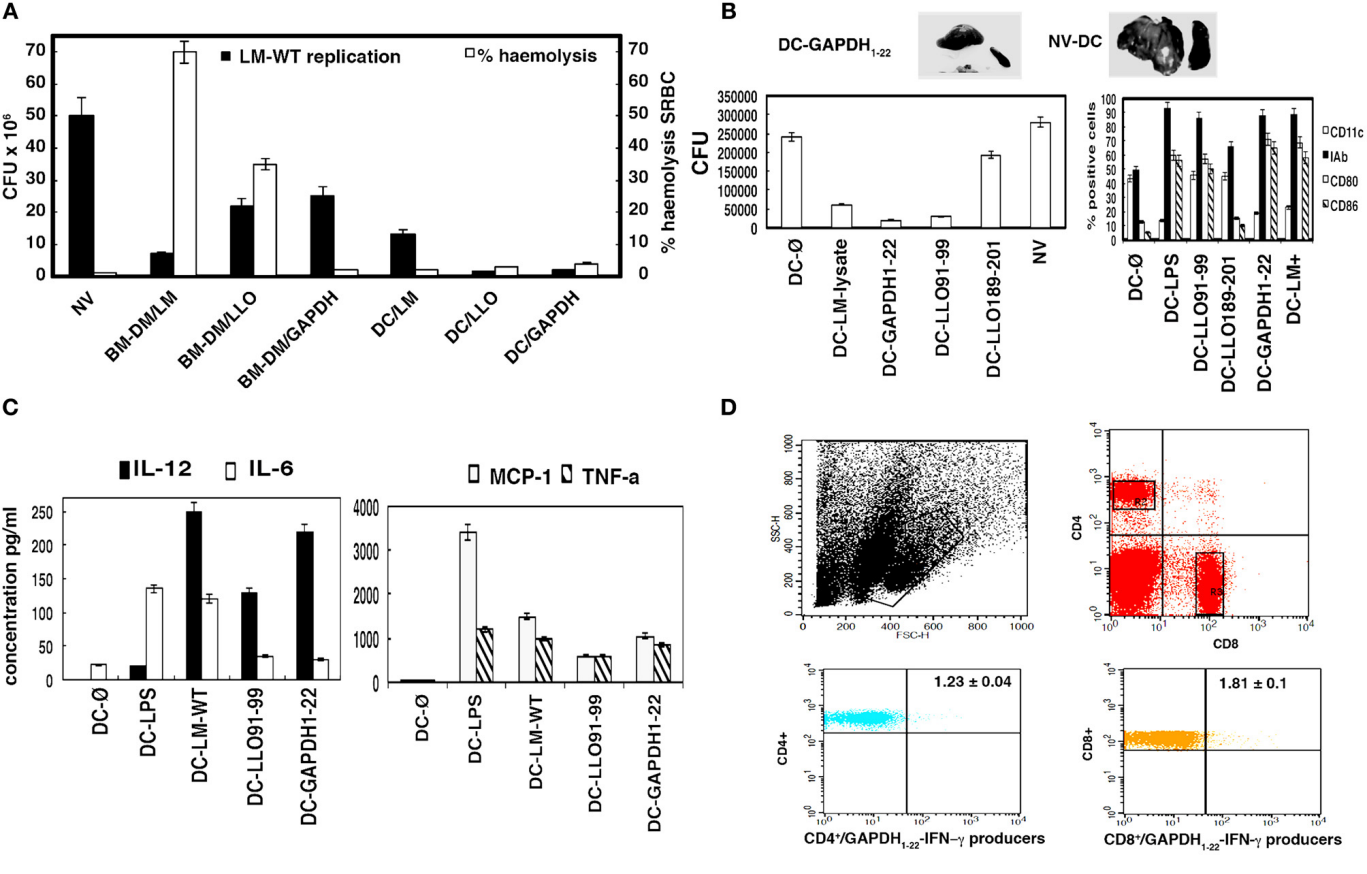
**Comparison of different BMDM and DC vaccine vectors for protection against listeriosis. (A)** C57BL/6 mice were vaccinated i.p. or not (NV) for 7 days with different BMDM or DC vaccine vectors (BMDM-LM-WT, BMDM-LLO, BMDC-LM-WT, BMDC-LLO, and BMDC-GAPDH) (1 × 10^6^ cells) (*n* = 5 mice/vaccine vector) and challenged i.p. with 5 × 10^3^ CFU LM-WT for 3 days. Results of spleens homogenates are mean ± *SD* of three different experiments (*P* < 0.01) (black bars in figure and left scale legend). Same vaccine vectors were incubated *in vitro* with SRBC (0.5% solution). Hemolytic units were defined as the dilution of the sample that caused 50% hemoglobin release from 200 ml of 0.5% SRBC. Controls were established for 0% hemolysis using empty DCs and for 100% hemolysis by incubating SRBC with distilled water. **(B)** C57BL/6 mice were vaccinated i.p. or not with empty DCs (DC-Ø) or with saline (NV) using the vaccine vectors: DC-LM lysate, DC-GAPDH_1−22_, DC-LLO_189−201_, DC-LLO_91−99_. We also vaccinated with the following control vectors: DC-LMWT, DC-LPS-LLO_91−99_, and DC-LPS-GAPDH_1−22_ protection results were as follows: 70, 92, and 98%. In several samples, positive selection of DC was performed with anti-mouse CD11c-coated magnetic beads and MACSTM separation columns, protection results of DC-LLO_91−99_ and DC-GAPDH_1−22_ after positive selection were as follows, 90 and 98%, respectively. Left plot corresponds to results of spleens homogenates that are the mean ± *SD* of three different experiments (*P* < 0.05). Surface expression of different markers was analyzed by FACS in the prepared DC vaccine vectors. Samples were acquired using FACSCanto flow cytometer and percentages of positive cells for each antibody are shown. Results are expressed as the mean ± *SD* of triplicate samples (*P* < 0.05). **(C)** Cytokines were measured in serum from vaccinated and NV mice (DC-Ø) from **(B)**. We also included another DC vaccine vector (DC-LPS) as a positive control. Levels of proinflammatory cytokines (MCP-1, TNF-α, IFN-γ, IL-6, IL-12, or IL-10) were analyzed by using the CBA kit (Becton Dickinson) and flow cytometry. Results were expressed as cytokine concentration (pg/ml of mean ± *SD*, *P* < 0.05). IL-12 concentration for DC-Ø vaccination was 8 ± 0.03 pg/ml and for DC-LPS, 19 ± 0.2 pg/ml. IFN-γ concentrations of samples were as follows: DC-LLO_91−99_ (389 ± 12 pg/ml), DC-GAPDH_1−22_ (425 ± 11 pg/ml), DC-LMWT (178 ± 10 pg/ml), DC-LPS (200 ± 0.3 pg/ml). **(D)** Spleen cells obtained from homogenates after vaccination were stimulated for 5 h with GAPDH_1−22_ in the presence of brefeldin A for intracellular cytokine staining. GAPDH-peptide-stimulated spleen cell surface was stained for CD4 or CD8 and fixed and permeabilized using a cytofix/cytoperm kit. Stimulated cells were surface stained for CD4 or CD8 using anti-CD4^+^ FITC-labeled or anti-CD8^+^ APC-labeled and data gated to include exclusively CD4^+^ or CD8^+^ events, R2, and R3 gates, respectively. Flow histograms show the percentages of GAPDH_1−22_/CD4^+^ and IFN-γ producers (lower left) and GAPDH_1−22_/CD8^+^ and IFN-γ producers (lower right) (R2 and R3 gates). Experiments were performed in triplicate and results are expressed as the mean ± *SD* (*P* < 0.05).

As a first approach using cellular vaccine vectors against listeriosis, we prepared different BMDM and DC vaccine vectors containing LM-WT, LLO, or GAPDH, and used them in vaccination protocols against an *L. monocytogenes* challenge of 5 × 10^3^ bacteria/mice (*n* = 5), inoculated for 3 days. We also tested the *in vitro* cytotoxicity of these vaccine vectors using a hemolysis assay with sheep-red blood cells, SRBC. Although BMDMs infected with LM-WT showed a good protection range, they also showed a high level of hemolysis (~70%). Two of five mice vaccinated died and showed hemolyzed livers (data not shown). BMDMs loaded with LLO showed ~50% protection but also 30% hemolysis, and one of five vaccinated mice died and presented with a hemolyzed liver (Figure 3A, gray bars). BMDMs loaded with GAPDH lacked cytotoxicity but their protection was low (~40%). We obtained the highest protection and the lowest cytotoxicity using DC-LLO or DC-GAPDH with 90–95% protection. DCs infected with LM-WT also showed good protection in the range of 65–70%, with no cytotoxicity (Figure 3A, compare black and gray bars, respectively). These results revealed that BMDMs were unsafe vaccine vectors against listeriosis, because they resulted in high cytotoxicity, either containing LM-WT or purified LLO. DCs seemed to be the safest vaccine vectors, conferring the highest protection against listeriosis.

To examine whether DCs loaded with LLO or GAPDH epitopes might also confer protection against listeriosis, we prepared DC vaccines loaded with peptides, DC-LLO_91−99_, DC-LLO_189−201_ or DC-GAPDH_1−22_, and compared them for protection with DCs loaded with an *L. monocytogenes* lysate (DC-LM lysate). Peptides in solution administrated as DC vaccines showed no protection at all (data not shown). As shown in Figure 3B (left plot), DC-GAPDH_1−22_ showed the highest protection (~99%), followed by DC-LLO_91−99_ (~94%) (Figure 3B, GAPDH_1−22_/DC and LLO_91−99_/DC bars in left plot). However, DC-LLO_189−201_ conferred little protection (~5%), comparable to DC empty vaccine vectors or mice inoculated with saline (Figure 3B, NV-DC bars in left plot). NV mice showed characteristic features of listeriosis, such as granulomatous lesions in the liver and splenomegalia (Figure 3B, NV-DC upper image). Vaccinated mice with DC-GAPDH_1−22_ showed a clear reduction in liver granulomatous lesions and presented normal size spleens (Figure 3B, DC-GAPDH_1−22_ image), similar to vaccination with DC-LLO_91−99_ (Kono et al., 2012). To note that positive selection of differentiated DCs with anti-mouse CD11c-coated magnetic beads and MACSTM separation columns did not give better protection results than our method (data shown in legend of Figure 3).

Effective DCs vaccine vectors present high percentages of positive CD80^+^ or CD86^+^ cells as classical DC activation markers as previously reported for DCs incubated with certain peptides (Pion et al., 2010). Therefore, we evaluated whether the high efficiency of DC-LLO_91−99_ or DC-GAPDH_1−22_ might correspond with high numbers of positive cells for these DC activation markers. As it is shown in Figure 3B (right plot), only DC-LLO_91−99_ or DC-GAPDH_1−22_ showed an increase in the number of positive CD80^+^ or CD86^+^ cells, similar to percentages obtained with DCs loaded with lipopolysaccharide, LPS (DC-LPS), used as a positive control. We also observed that DC-GAPDH_1−22_ showed a decrease in the percentages of positive CD11c^+^ cells, similar to the pattern of DC-LPS, while DC-LLO_91−99_ showed no modification on the percentages of positive CD11c^+^ cells. A decrease in the number of positive CD11c^+^ cells in DCs appears linked to polarized DCs, as in the case of LPS-loaded DCs (Kono et al., 2012).

We confirmed whether the protection capacity of DC vaccine vectors was linked to Th1 cytokine production. As shown in Figure 3C, DC vaccine vectors conferring protection against listeriosis, such as DC-LM-WT, DC-LLO_91−99_ or DC-GAPDH_1−22_, produced high levels of interleukin (IL)-2 and IFN-γ (570–589 ± 0.9 pg/ml, and NV mice basal levels, 0.5 ± 0.02 pg/ml) and significant levels of monocyte chemotactic protein (MCP)-1 and tumor necrosis factor (TNF)-α. Mice vaccinated with DC-GAPDH_1−22_ produced significantly more IL-12 than those vaccinated with DC-LLO_91−99_ did. Production of IL-6 was significantly reduced after vaccination with DC-LLO_91−99_ or DC-GAPDH_1−22_, indicating that the classical acute inflammation in listeriosis was avoided with vaccination. Therefore, the vaccination efficiency of DC-GAPDH_1−22_ was related to high production of IL-12, as well as significant levels of other Th1 cytokines such as IFN-γ, MCP-1, and TNF-α. We did not observe any production of IL-10 in DC-vaccinated mice.

Vaccine efficiency is also linked to DC ability to expand the immune response, stimulating different T cells, both CD4 and CD8 subsets. Therefore, we analyzed the spleens of mice vaccinated with DC-LLO_91−99_, DC-LLO_189−201_, or DC-GAPDH_1−22_ for production of a primary T cell response. We observed 1.2 ± 0.05% positive CD8^+^-restricted LLO_91−99_ cells and IFN-γ producers after vaccination with DC–LLO_91−99_. We also observed 0.75 ± 0.02% positive CD4^+^-restricted LLO_189−201_ cells and IFN-γ producers after vaccination with DC-LLO_189−201_. We observed 1.23 ± 0.04% CD4^+^-restricted GAPDH_1−22_ cells and IFN-γ producers and 1.81 ± 0.1% CD8^+^-restricted GAPDH_1−22_ cells and IFN-γ producers (Figure 3D). These results suggested that although vaccination with DC-LLO_189−201_ induced a primary and specific CD4^+^ T cell response, these vaccines did not confer any protection against listeriosis. Vaccination with DC-LLO_91−99_ induced a strong and specific CD8^+^ T cell response and conferred protection. Vaccination with DC-GAPDH_1−22_ induced specific CD4^+^ and CD8^+^ T cell responses and was highly protective.

We confirmed the frequency of LLO_91−99_-specific and GAPDH_1−22_-specific CD8^+^ T cells using H2-K^b^-LLO_91−99_ or H2-K^b^-GAPDH_1−22_ dimers by flow cytometry after vaccination with DC-LM-WT, DC-LLO_91−99_ or DC-GAPDH_1−22_. As shown in Table 3, the occurrence of LLO_91−99_-specific CD8^+^ cells producing IFN-γ after vaccination with DC-LLO_91−99_ was 1.22% of positive gated cells, which was higher than the frequency observed with DC-LM-WT (0.48%) (*P* < 0.05). The occurrence of GAPDH_1−22_-specific CD8^+^ cells was 4.02% of positive gated cells; which was higher than the frequency one observed with DC-LM-WT-vaccinated mice (1.53%). Therefore, primary T cell responses highlighted the role of *L. monocytogenes*-specific CD8^+^ T cells to confer significant protection against listeriosis, while CD4^+^ T cells seemed to participate less in protection.

**Table 3:** **Frequencies of LLO_91−99_- and GAPDH_1−22_-specific CD8^+^ T cells induced by DC vaccines**.

**Vaccination type**	**% Total dimer-CD8/LLO_91-99_**	**% Gated dimer-CD8/LLO_91-99_**	**% Total dimer-CD8/GAPDH_1-22_**	**% Gated dimer-CD8/GAPDH_1-22_**
DC-LM-WT	0.05 ± 0.001	0.48 ± 0.01	0.05 ± 0.002	1.53 ± 0.02
DC-LLO_91−99_	0.06 ± 0.001	1.22 ± 0.02	NA	NA
DC-GAPDH_1−22_	NA	NA	0.16 ± 0.001	4.02 ± 0.03

*Splenocytes from vaccinated mice were incubated with recombinant dimeric H-2K^b^:Ig fusion protein loaded with LLO_91-99_ or GAPDH_1-22_ peptides. The staining cocktail contained the dimeric fusion protein loaded with the peptides, CD8 and IFN-γ antibodies. CD8^+^ cells were gated for anti-IFN-γ staining (% Gated dimer-CD8) to calculate the frequencies of CD8^+^-LLO_91-99_ or CD8^+^-GAPDH_1-22_ restricted cells and IFN-γ producers. p < 0.05*.

## Discussion

Recently, cell-based vaccines have received considerable impetus following their success against infectious diseases and cancer (Starks et al., 2004; Kim et al., 2009; Pion et al., 2010; Singh et al., 2011; Kono et al., 2012; Martirosyan et al., 2012; Matsumura et al., 2013; Quispe-Tintaya et al., 2013; Vacas-Cordoba et al., 2013). In the case of listeriosis vaccination, the emphasis has been on recombinant vaccines using live attenuated pathogens, metabolically active but non-viable bacteria or non-pathogenic bacteria harboring virulence gene clusters, although some of them do not trigger strong innate immune responses and require adjuvants (Brockstedt et al., 2005; Bruhn et al., 2007; Mohamed et al., 2012). These recombinant vaccines might also present safety concerns because live vaccines if administered to immunocompromised individuals might cause cytotoxicity. However, cellular vaccines such as BMDMs or DCs, or subcellular vectors such as endosomes or phagosomes appear to be safer candidates to harbor *L. monocytogenes* antigens because the vaccinated individuals are not directly exposed to live bacteria (Sashinami et al., 2003; Rodriguez-Del Rio et al., 2011; Carrasco-Marín et al., 2012; Kono et al., 2012). Moreover, high phagocytic abilities and induction of strong innate and specific immune responses are features that make cellular vaccines attractive.

We have attempted to design novel cell-based vaccines for listeriosis lacking cytotoxicity and pathogenicity and showing high protective activity. For this purpose, we chose BMDMs and DCs as vaccine vectors against *L. monocytogenes*. In this regard, the features of the cellular vaccines conferring successful protection against listeriosis were high phagocytic activity and good antigen processing. We observed that BMDMs and DCs infected with LM-WT shared equivalent listericidal features, activation patterns, and antigen processing competency. Therefore, BMDMs and DCs showed similar *L. monocytogenes* growth kinetics and degradation of the *hly*-deficient mutant LM-ΔLLO. BMDMs were CD11b^+^F4/80^high^MHC-II^high^ cells and DCs were CD11c^+^F4/80^high^CD11b^low^MHC-II^high^ cells. BMDMs and DCs transformed *L. monocytogenes* phagosomes into MIIC compartments with similar ratios, showing 70–72% colocalization of GFP-LM with MHC-II molecules. Unexpectedly, protection and cytotoxicity of these vaccine vectors were different. BMDMs showed protection ratios lower than those of DCs and high cytotoxicity measured *in vitro* by SRBC hemolysis and *in vivo* by examining death in mice. Recently, LLO immunogenicity and cytotoxicity were dissociated, indicating that LLO was available in *L. monocytogenes* infection (Carrero et al., 2012). It is possible that different amounts of LLO were available for BMDMs or DCs or different surface receptors, explaining their differences in cytotoxicity. Therefore, we selected DCs as the vectors showing features of safe vaccines. We also searched for the most suitable *L. monocytogenes* antigen that triggered protective Th1 responses. Although CD8^+^ T cells recognize several epitopes, immunodominant CD8 epitopes are eluted from MHC-I molecules and associate with effector functions. Pathogen-specific CD8^+^ T cells play a major role in protection against listeriosis, but also pathogen-specific CD4^+^ T cells efficiently collaborate in conferring protective immunity (Skoberne and Geginat, 2002; Pamer, 2004; Nagata and Koide, 2010). To date, LLO has been revealed as the immunodominant antigen in listeriosis and the CD8^+^-specific LLO_91−99_ epitope is the major epitope eluted from MHC-I molecules, which confers protection (Kono et al., 2012). However, the CD4^+^-specific LLO_189−201_ epitope also confers protection against listeriosis and appears as the major epitope eluted from MHC-II molecules (Geginat et al., 2001; Skoberne and Geginat, 2002). In this study, we presented the abilities of a novel *L. monocytogenes* antigen, GAPDH, which is a powerful antigen that triggers T cell responses *in vivo* (Álvarez-Domínguez et al., 2008 and this study in the hind foot pads). We also described the features of the GAPDH_1−22_ peptide that contained CD8^+^-specific and CD4^+^-specific epitopes that were combined in DC vaccine vectors; DC-GAPDH_1−22_, confers protection against listeriosis with higher ratios than DC-LLO_91−99_ vectors. Several characteristics of GAPDH_1−22_ peptide make this long epitope attractive for vaccination purposes. GAPDH_1−22_ peptide was a strong binder to MHC-I and a weak binder to MHC-II molecules. This peptide contains at least two strong binding sequences to MHC-I molecules similar to LLO_91−99_ epitope, sequences GAPDH_5−15_ and GAPDH_8−18_. This peptide also shows two weak binding sequences to MHC-II molecules similar to LLO_189−201_ epitope, sequences GAPDH_4−18_ and GAPDH_8−22_. According to the 3D structure modeling, GAPDH_5−15_ is localized in a loop similar to the 3D loop structure of LLO_91−99_ and GAPDH_8−22_ in the α-helix structure that is shared by most MHC-II binding sequences, including LLO_189−201_. Combination of MHC-I and MHC-II binding epitopes might explain the ability of DC-GAPDH_1−22_ vaccine vector to confer higher protection against listeriosis than DC-LLO_91−99_ vaccine vector can. It can also explain why DC-GAPDH_1−22_ vaccine vector elicited greater primary T cell responses since it can elicit both, CD4^+^ and CD8^+^-restricted responses. However, DC-LLO_189−201_ and DC-LLO_91−99_ vaccine vectors elicit only CD4^+^ or CD8^+^-restricted responses, respectively. Moreover, these results are also in agreement with studies indicating that *L. monocytogenes*-specific CD4^+^ T cells played a role in protection against listeriosis, probably collaborating with immunodominant epitope-specific CD8^+^ T cells (Nagata and Koide, 2010). DC-GAPDH_1−22_ vaccine vectors produced higher IL-12 levels and frequencies of GADPH_1−22_-restricted CD8^+^ T cells than the induction observed with the single CD8^+^ epitope DC-LLO_91−99_ vaccine vector. Production of IL-12 is related to anti-*Listeria* responses and with vaccine success (Kono et al., 2012; Lee et al., 2013). Therefore, it seems that DC designs containing CD8^+^ and CD4^+^ epitopes such as DC-GAPDH_1−22_ are effective vaccine vectors. Production of Th1 cytokines MCP-1, TNF-α and IFN-γ and lack of production of Th2 cytokines such as IL-6 and IL-10, which are responsible for exaggerated inflammatory reactions and putative autoimmune responses in listeriosis, are features that are also relevant for protective DC vaccine vectors (Kono et al., 2012; Vacas-Cordoba et al., 2013).

In summary, the present study revealed a novel DC design, DC-GAPDH_1−22_, which confers greater protection against listeriosis than the previously described DC design, DC-LLO_91−99_. We conclude that DC-GAPDH_1−22_, which combines several CD4^+^ and CD8^+^ epitopes in a single vaccine vector, shows greater protection against listeriosis, because this DC vaccine increases the magnitude of primary T cell responses, the percentage of GAPDH_1−22_-restricted CD8^+^ T cells, and the levels of IL-12. Also, the phenotype of DC-GAPDH_1−22_ vaccine vector with higher expression of the maturation markers, CD80 and CD86, characteristic of polarized and IL-12-producing DCs (Kono et al., 2012) might also contribute to protective efficacy.

### Conflict of interest statement

This study is hold by Spanish patent 200602175(3) and the international extend PCT/ES2007/070144. These patents are licensed to Fundación Marqués de Valdecilla and the authors are Carmen Álvarez-Domínguez and Eugenio Carrasco-Marín. The other authors declare that the research was conducted in the absence of any commercial or financial relationships that could be construed as a potential conflict of interest.
